# Cortical laminar necrosis triggered by hepatic encephalopathy and post-traumatic subarachnoid hemorrhage: Case report

**DOI:** 10.1016/j.radcr.2024.11.082

**Published:** 2024-12-18

**Authors:** Inas AL CHARE, Mickaël BONNAN

**Affiliations:** Department of Neurology, Delafontaine Hospital, Seine Saint-Denis, France

**Keywords:** Cortical laminar necrosis, Hepatic encephalopathy, Subarachnoid hemorrhage, Case report

## Abstract

Hepatic encephalopathy may trigger cortical laminar necrosis (CLN), which is characterized by diffuse symmetric cortical lesions. We report a 56-year-old woman with liver cirrhosis who presented with prolonged floor station, reduced alertness and left hemiplegia. Blood ammonia level was elevated. Magnetic resonance imaging showed acute left cerebellar infarction, diffuse cortical lesions mostly involving the right temporo-fronto-parietal cortex with restricted diffusion, and right subarachnoid hemorrhage. This case of CLN lesions related to hepatic encephalopathy is characterized by unique pattern of almost unilateral lesions. We hypothesize that the right predominance of the brain cortical lesions could be a consequence of synergistic factors associating subarachnoid hemorrhage in the setting of hepatic encephalopathy. This finding refines the pathophysiology of metabolic CLN.

## Introduction

Cortical laminar necrosis (CLN) is the pseudo-laminar spongiform degeneration of the deep layers of the cortex, with the presence of Alzheimer 2 type astrocytes in the cortex and basal ganglia. These cells display a large pale nucleus, marginal chromatin, and scanty cytoplasm. Typically, magnetic resonance (MR) imaging in the acute phase shows a T2-weighted(w) hyperintense signal in the cortical areas. The chronic phase is characterized by a T1w hyperintense signal in the cortex and cortical atrophy. CLN may be a complication of various disorders: mainly hypoxic encephalopathy, but also metabolic, infectious, and toxic encephalopathy. The pattern of lesions depends on the causes [1]. Metabolic triggers are typically associated with bilateral and symmetric lesions. Only few cases of CLN extending to the bilateral fronto-temporo-parietal cortex have been described in association with hepatic encephalopathy. This unique case of almost unilateral CLN associated with hepatic encephalopathy unmasks the local role of synergistic metabolic triggers.

## Case report

A 56-year-old woman was admitted for reduced alertness. The neurologic examination revealed left hemiplegia, left homonymous hemianopsia, and a rightward gaze. She was pauci-communicative. Temperature was 32.9°C, without any sign of cardiac or respiratory failure. There was a left parietal subcutaneous hematoma.

**Fig. 1:**
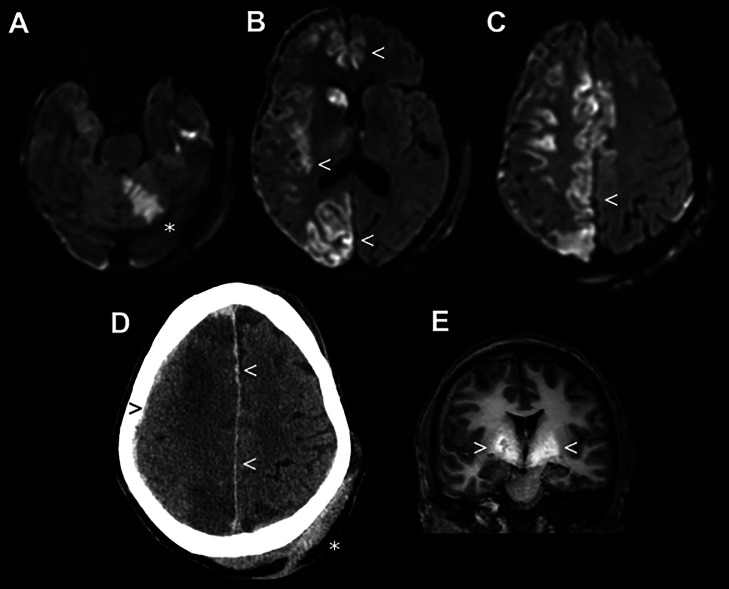
Cortical laminar necrosis (CLN) on brain MRI. (A) Acute left cerebellar stroke (*), which is the putative explanation why the patient fell and stayed down. Other brain lesions, which spared the white matter, were not congruent with arterial territories. Willis circle, brain and cervical arteries were normal (not shown). (B and C) Diffuse CLN spread over the right hemisphere and minimally diffusing over the left paramedial cortex (<). (D) Cranial computed tomography showing left subgaleal traumatic hematoma (*) and right contrecoup subdural (>) and subarachnoid hemorrhage (<). **(**E) Increased T1w signal in deep grey matter structures (>), typically associated with chronic hepatic encephalopathy. Axial brain MRI with DWI (A-C) and T1-weighted (E) sequences.

Past medical history revealed HIV infection, which was well-controlled under tri-therapy, and liver cirrhosis with a history of hepatic encephalopathy requiring a transjugular intrahepatic porto-systemic shunt (TIPS). The liver function test revealed moderate hepatic cytolysis. Serum electrolytes and blood glucose levels were within normal ranges. Blood culture, alcohol and drug tests were negative. Diffusion-weighted imaging (DWI) revealed acute left cerebellar infarction and diffuse cortical lesions, mostly involving the right temporo-fronto-parietal cortex including the caudate nucleus (Fig. 1). Diffusion on the apparent diffusion coefficient (ADC) map was restricted. The left fronto-parietal cortex was minimally involved. Similar lesions were observed on a fluid-attenuated inversion recovery (FLAIR) sequence. T1-weighted images showed a high-intensity signal in both globi pallidi, which is a sign of chronic hepatic encephalopathy. MR images also revealed a right subarachnoid hemorrhage and a predominantly right-sided subdural hematoma.

Electroencephalogram (EEG) displayed generalized slowing consistent with encephalopathy. It was more pronounced in the right hemisphere and was attributed to right subarachnoid hemorrhage. Blood ammonia level was still 85 µM (normal range 12-47 µM) a few days after admission.

The fall could have been caused by the cerebellar stroke, and complicated by head trauma, as suggested by the left parietal subcutaneous hematoma. The backlash injury had caused a predominantly right-sided subarachnoid and subdural hemorrhage. Hepatic encephalopathy was triggered by the prolonged ground station, explaining the high level of ammonia in the blood. Additionally, abdominal computed tomography showed TIPS thrombosis. We concluded that the CLN occurred in the context of hepatic encephalopathy, as evidenced by sparing of the peri-rolandic and occipital cortices on MRI images. Extensive subarachnoid hemorrhage may also have triggered localized CLN. This unique asymmetric pattern of CLN may be due to this dual etiology.

The patient's recurrent impaired awareness supported the hypothesis of generalized seizures, although EEG data did not confirm this. Nevertheless, she received levetiracetam and valproate. Valproate reduced her level of alertness and EEG demonstrated triphasic waves, which abated after discontinuing valproate. After 1 month of intensive care, she recovered normal consciousness but severe clinical signs of right-sided lesions persisted.

## Discussion

Most reported cases of CLN occur in a context of hypoxic encephalopathy, and only rare cases are triggered by hepatic encephalopathy. CLN due to hypoxic encephalopathy is mainly observed in the parieto-occipital cortex owing to hemodynamic factors [2].

Classically, hyperammonemic encephalopathy due to hepatic failure always occur with bilateral and symmetric distribution [[3], [4], [5], [6], [7]]. Our patient showed extensive cortical lesions on DWI sequences and restricted diffusion on the ADC map. However, no episode of hypotension or hypoxemia were reported and only cerebellar lesions could be explained by an ischemic event. The right temporo-fronto-parietal cortex was electively affected while the peri-rolandic and occipital cortices were spared. Although, unilateral CLN lesions may reveal inherited metabolic disorders, this case was considered highly improbable.

These MRI findings are thought to be due to toxic ammonia levels leading to astrocytic swelling. Moreover, high blood ammonia levels induce cell toxicity and elevated glutamine levels by reducing the synaptic clearance of glutamate released by neurons. Extracellular glutamate has epileptogenic and excitotoxic effects, and patients suffering from hepatic encephalopathy may develop prolonged focal seizures [8], although blood ammonia levels remained minimally increased (below 140 µmol/L) in our patient.

Prolonged seizures are known to enhance CLN [8], probably due to excitotoxic mechanisms, and may explain asymmetric lesions. However, our patient did not undergo any seizure, EEG mostly revealed signs of encephalopathy, and the pulvinar nuclei were unaffected. Valproate tends to increase ammonia blood levels even when liver function is normal, so its use in hepatic encephalopathy requires caution.

Lastly, unlike the typically symmetrical cortical lesions described in hyperammonemic encephalopathy, the MRI features in our case were highly asymmetric with obvious right-sided dominance. This finding may be triggered by the backlash subarachnoid hemorrhage, which was restricted to the right hemisphere. Indeed, focal delayed CLN has been rarely reported after traumatic subarachnoid hemorrhage [9]. In animal models, hemolysis in the subarachnoid space leads to angiographic vasospasm with ischemia spreading to the adjacent tissues, which finally may trigger CLN [10,11]. Thus, in our case, we assume that CLN was triggered by hepatic encephalopathy and was exacerbated in the right hemisphere due to subarachnoid hemorrhage.

## Conclusion

This unique case of asymmetric metabolic CLN was triggered by hepatic encephalopathy combined with subarachnoid hemorrhage lesions. This finding shows that combined etiologies can facilitate the appearance of CLN. This finding refines the pathophysiology of metabolic CLN.

## Ethics

This work was conducted in accordance with French Ethics guidelines in noninterventional studies with anonymized data.

## Patient consent

Informed consent was obtained from the patient.
